# Relationship between functional network integrity, cognition and fatigue at long-term follow-up in patients with IDH-mutated gliomas

**DOI:** 10.3389/fnhum.2026.1860966

**Published:** 2026-07-16

**Authors:** Gaia Olivo, Isabelle Linnea Rydén, Alice Neimantaite, Erik Thurin, Asgeir Store Jakola

**Affiliations:** 1 Department of Psychology, University of Gothenburg, Gothenburg, Sweden; 2 Department of Clinical Neuroscience, Sahlgrenska Academy, University of Gothenburg, Gothenburg, Sweden; 3 Department of Neurology, Sahlgrenska University Hospital, Gothenburg, Sweden; 4 Department of Radiology, Sahlgrenska University Hospital, Gothenburg, Sweden; 5 Department of Neurosurgery, Sahlgrenska University Hospital, Gothenburg, Sweden

**Keywords:** brain networks, brain plasticity, executive function, glioma, MRI

## Abstract

**Background:**

Many patients with *IDH*-mutated gliomas struggle with cognitive dysfunction and mental fatigue at long-term follow-up. Here, we investigate the relationship between functional network re-organization and long-term cognitive outcomes in patients treated for *IDH* mutated glioma.

**Methods:**

We examined 61 patients at 5–21 years post-surgery using resting-state fMRI and neuropsychological testing. Individual-level network maps were obtained by regressing each patient’s resting-state time series against reference template networks via dual regression, and network integrity was quantified as the spatial correspondence between these maps and the corresponding healthy templates. The Mental Fatigue Scale and Hospital Anxiety and Depression Scale were used to assess fatigue, depression and anxiety.

**Results:**

Tests of executive function, processing speed/attention, and language showed significant associations with auditory, executive control, and frontoparietal network-template similarity. Memory domains displayed distinct patterns: visual memory correlated with network-template similarity of the default mode network, and verbal memory with the auditory network. Mental fatigue correlated with executive function, processing speed/attention, and language performance. Three cognition-fatigue profiles emerged, distinguished by depression/anxiety levels and auditory/sensorimotor network integrity.

**Conclusion:**

In patients with IDH mutated gliomas at long-term follow-up, preserved cognitive performance is associated with functional network organization resembling healthy brain templates. For patients with cognitive impairment, different networks were linked to distinct cognitive domains. Patients with a combination of mental fatigue and executive impairment were characterized by most pronounced network re-organization and highest symptoms of anxiety and depression. These findings suggest network-specific rehabilitation approaches targeting the interplay between fatigue, mood, and executive control may improve patient outcomes.

## Introduction

Gliomas constitute approximately 80% of all malignant brain tumors, and are classified according to the 2021 World Health Organization (WHO) classification system (Board WCoTE, 2021). The integration of molecular markers, particularly isocitrate dehydrogenase (*IDH*) mutation status, has revolutionized glioma classification, as infiltrative gliomas with *IDH*-mutation tumors generally have more favorable outcomes compared to their *IDH*-wildtype counterparts (Board WCoTE, 2021). Treatment approaches are multimodal, typically involving surgical resection, radiotherapy, and chemotherapy (Sledzinska et al., 2023; Weller et al., 2021). However, long-term survivors remain under-investigated, and the neural correlates of their cognitive and fatigue profiles are poorly understood.

Cognitive impairment represents one of the most debilitating consequences of gliomas (Kirkman et al., 2022; Ng et al., 2019; Tanzilli et al., 2022), and can occur across multiple domains, substantially reducing quality of life (Kirkman et al., 2022; Ng et al., 2019; Tanzilli et al., 2022). A large prospective longitudinal study following 130 patients over 24 months demonstrated that executive function, along with long- and short-term memory, were the most frequently affected domains (Tanzilli et al., 2022). Furthermore, progressive cognitive decline is common, occurring in approximately one-third of patients during follow-up (Tanzilli et al., 2022). The factors underlying individual differences in cognitive trajectories remain poorly understood. While tumor characteristics (including location, laterality, volume, lesion momentum (Wefel et al., 2016), and grade) and treatment effects serve as primary determinants (Kirkman et al., 2022), additional factors such as brain reserve, sex, comorbidities, mood states, and mental fatigue can further influence this relationship (Kirkman et al., 2022). Moreover, while mental fatigue can impair cognitive performance on sustained attention, working memory, executive control, and processing speed (Gavelin et al., 2023), subjective mental fatigue and objective performance measures are frequently dissociated (Van Dongen et al., 2011), suggesting that perceived effort and actual cognitive capacity represent partially independent dimensions of mental fatigue (Van Dongen et al., 2011).

Resting-state functional magnetic resonance imaging (rs-fMRI) has emerged as a valuable tool for investigating brain network organization and for surgical planning in patients with brain tumors (Sparacia et al., 2021; Metwali and Samii, 2019; Sighinolfi et al., 2022), potentially serving as a marker for radiotherapy- and surgery-induced cognitive damage (Liu et al., 2025; Tran et al., 2024). Rs-fMRI measures spontaneous brain activity while participants are at rest (i.e., without performing specific tasks). It offers advantages over task-based fMRI including easy clinical implementation, feasibility in severely impaired patients, and rich networks characterization (Metwali and Samii, 2019; Sighinolfi et al., 2022; Maniar et al., 2021).

Despite inherent heterogeneity in tumor locations and sizes, several studies have employed rs-fMRI to examine the complexity of network reorganization in glioma patients. Differences have been reported primarily in the default mode network (DMN), dorsal attention network, fronto-parietal network (FPN), and cerebellum, compared with controls (Sighinolfi et al., 2022; Tordjman et al., 2021). Network modifications are location-specific (Pasquini et al., 2022), with frontal and temporal tumors showing more extensive bilateral changes than parietal and insular tumors (Pasquini et al., 2022). Moreover, functional remodeling appears hemisphere-specific and is predicted by tumor grade (Cai et al., 2020). Neuroimaging studies using rs-fMRI have also attempted to elucidate the mechanisms underlying executive deficits in glioma patients, though their neural determinants remain poorly understood. Alterations in DMN, FPN, salience, and executive control networks have been related to cognitive alterations (Liu et al., 2019; Yang et al., 2021; van Lingen et al., 2023), with both network integrity and cognitive recovery depending on tumor location, structural brain changes, and education rather than demographic or treatment variables (Kocher et al., 2020; Harris et al., 2014; Saviola et al., 2022). Functional connectivity between tumor-infiltrated tissue and attention networks also correlates with better long-term cognitive outcomes, indicating that infiltrated tissue can still participate in cognitive circuits (Mandal et al., 2024).

Several significant limitations persist in the current literature. Most studies have small sample sizes, limiting statistical power and generalizability. Follow-up studies typically span only one to few years post-surgery, limiting our understanding of long-term cognitive trajectories and network reorganization in glioma survivors. Only one pilot study examined long-term survival (up to 10 years, *n* = 22) with rs-fMRI, finding stronger connectivity in medial prefrontal cortex and intraparietal sulcus among cognitively non-impaired versus impaired survivors (Wang et al., 2022). However, this study’s small sample size and pilot nature highlight the critical need for larger, more comprehensive investigations.

The aim of this study is therefore to examine how resting-state network organization relates to domain-specific cognitive abilities in 61 long-term glioma survivors with the most extensive long-term follow-up to date (up to 21 years).

## Methods

### Participants

Recruitment was conducted between August 19, 2022, and November 20, 2024, at Sahlgrenska University Hospital, Gothenburg, Sweden. In total, 69 patients who had undergone biopsy or resective surgery and had a histopathologically verified IDH mutated WHO grade 2–4 glioma according to the 2021 WHO classifications of tumors in the central nervous system (Board WCoTE, 2021) at least 5 years earlier (range 5–21 years; mean: 8.4 years; standard deviation (SD): 7.4) were invited to participate. Patients had to be at least 18 years old at the time of diagnosis. Exclusion criteria included non-IDH-mutated tumor status and inability to complete cognitive testing. Comorbidities and medication use were systematically screened and recorded for all participants, but were not considered as exclusion criteria due to the high prevalence of comorbidities in patients with gliomas.

Four patients declined participation, and two patients were missing MRI data and were therefore excluded from the analysis. 63 patients with gliomas were enrolled in the study. fMRI data were not usable for two patients (see below); therefore, the total sample consisted of 61 patients. Neuropsychological assessment and MRI were conducted within a median interval of 0 days (Q1–Q3: 0–26 days); all patients were free of tumor recurrence or progression during the period between assessments, as confirmed by clinical and radiological review. Structural MRI, resting-state fMRI, and neuropsychological testing were acquired.

The age at follow-up ranged between 25 to 80 years (mean: 48 years; SD: 11.3). Comorbidities included: obesity (*n* = 2), hypertension (*n* = 3), hyperlipidemia (*n* = 2), type 1 diabetes (*n* = 1), type 2 diabetes (*n* = 1), anxiety requiring pharmacological treatment (*n* = 2), stroke (*n* = 2), treated hypothyroidism (*n* = 2), obstructive sleep apnea (*n* = 2), chronic obstructive pulmonary disease (COPD) (*n* = 1), previous cancer (*n* = 1), and previous hysterectomy (*n* = 1). Antiepileptic drugs (AEDs) were used by 64% of the sample.

### Neuropsychological assessment

Neuropsychological assessments were conducted by a clinical neuropsychologist (I.R.). Prior to the assessment, patients were asked to complete self-report questionnaires and bring them to the appointment. The self-assessment included the Hospital Anxiety and Depression Scale (HADS), used to screen for symptoms of anxiety and depression. A score above 8 on either subscale was considered indicative of clinically relevant symptoms (Wu et al., 2021). Mental fatigue was evaluated using the Mental Fatigue Scale (MFS), applying a clinical cut-off of 10.5 points.

The neuropsychological test battery comprised the following instruments: Brief Visuospatial Memory Test–Revised (BVMT-R), Rey Auditory Verbal Learning Test (RAVLT), Rey Complex Figure Test (RCFT), Boston Naming Test (60-item version; BNT), Delis–Kaplan Executive Function System (D-KEFS), Verbal Fluency (phonemic fluency and semantic fluency: animals), Wechsler Adult Intelligence Scale (WAIS)-IV Digit Span (forward and backward), WAIS-IV Coding, Trail Making Test parts A and B (TMT A and B), and the D-KEFS Color Word Interference Test (CWIT), conditions 1–4. A detailed description of the tests can be found in Supplementary Table S1. To minimize practice effects, parallel test versions were used when available (RAVLT and BVMT-R), with randomization of starting versions to reduce order bias.

### Image acquisition

MRI scanning was performed on a Philips MR 7700 3 Tesla scanner with 32-channel head coil (Best, the Netherlands) at Sahlgrenska University Hospital in Gothenburg, Sweden. Anatomical images of the brain were acquired with 3D T1-weighted turbo-field-echo sequence (slice thickness: 1 mm; slice spacing: 1 mm; repetition time (TR) = 8.3 ms; echo time (TE) = 3.8 ms; flip angle = 8°; voxel size = 1 × 1 × 1 mm^3^) and a FLAIR (Fluid-Attenuated Inversion Recovery) sequence (slice thickness: 1.12 mm; slice spacing: 0.56 mm; TR = 8 s; TE = 0.3 s; TI = 2.4 s; flip angle = 90°; voxel size = 0.56×0.74×0.74 mm^3^). Resting-state data were acquired with a T2-weighed echo-planar imaging (EPI) sequence, collecting 300 volumes with 60 slices with ascending order, using a field of view of 108 × 110 mm and isotropic voxels of 2 × 2 × 2 mm^3^. Slice thickness was set at 2 mm, and 2.2 mm gap between the interleaved scans (TR = 1.5 s, TE = 30 ms; flip angle = 70°).

### Structural images processing

Structural images underwent a multi-step preprocessing pipeline designed to handle brains with lesions. First, lesion segmentation was performed using the “LST: Lesion Segmentation Tool”^1^ for Statistical Parametric Mapping (SPM) 12.^2^ The lesion prediction algorithm implemented in the Clinical Toolbox was used to obtain automated segmentation of residual lesions (due to residual/recurrent tumor and/or therapy-induced damage) present in each subject’s brain based on FLAIR images, resulting in the generation of subject-specific lesion masks. The T1-weighted images underwent unified segmentation and normalization to Montreal Neurological Institute (MNI-152) (Mazziotta et al., 2001) standard space, with the lesion masks used to exclude lesion regions from tissue probability calculations. This approach prevents lesioned tissue from being misclassified as normal brain tissue during segmentation. Probability maps for grey matter, white matter, and cerebrospinal fluid were visually inspected to ensure adequate quality of the registration and normalization procedures, with particular attention to regions adjacent to lesions. Finally, normalized structural images were smoothed with an 8 mm full width at half maximum (FWHM) Gaussian kernel to increase signal-to-noise ratio and account for residual inter-subject variability remaining after the normalization procedure.

### Functional images processing

Preprocessing of functional images was carried out in SPM12, running on Matlab R2023b. Slice timing correction was first applied to adjust for temporal delays in the acquisition of different slices within each volume. Realignment was then performed to correct for head motion, using the first acquired image as a reference. Two patients had moved more than 3 mm and were excluded from further analyses. Co-registration was performed to align functional images with each subject’s FLAIR image, then functional images were then normalized to MNI standard space using the same transformation parameters derived from the structural normalization. Normalized images were visually inspected to ensure the good quality of the coregistration and normalization procedures. Images were then smoothed with 4 mm FWHM Gaussian kernel. Finally, band-pass filtering was applied to the functional scans (0.01–0.1 Hz) to remove residual motion and physiological artefactual effects from the BOLD signal.

Following pre-processing, network parcellation was carried out with dual regression in FSL (FMRIB Software Library) package (Jenkinson et al., 2012) on the brain-maps resting-state networks provided by Smith et al. (2009), and available on the FMRIB’s website.^3^ Dual regression is a two-stage technique that allows subject-level spatial maps and associated time series to be derived from a set of group-level independent components. In the first stage, the group-level spatial maps are used as a set of regressors in a multiple regression against each subject’s 4D dataset, yielding one time series per component per subject. In the second stage, these time-series are in turn used as regressors against the same 4D dataset, producing spatial maps for each network for each subject. This approach allows between-subject variability in network topology to be captured while maintaining correspondence to a common set of reference networks.

**Figure 1:**
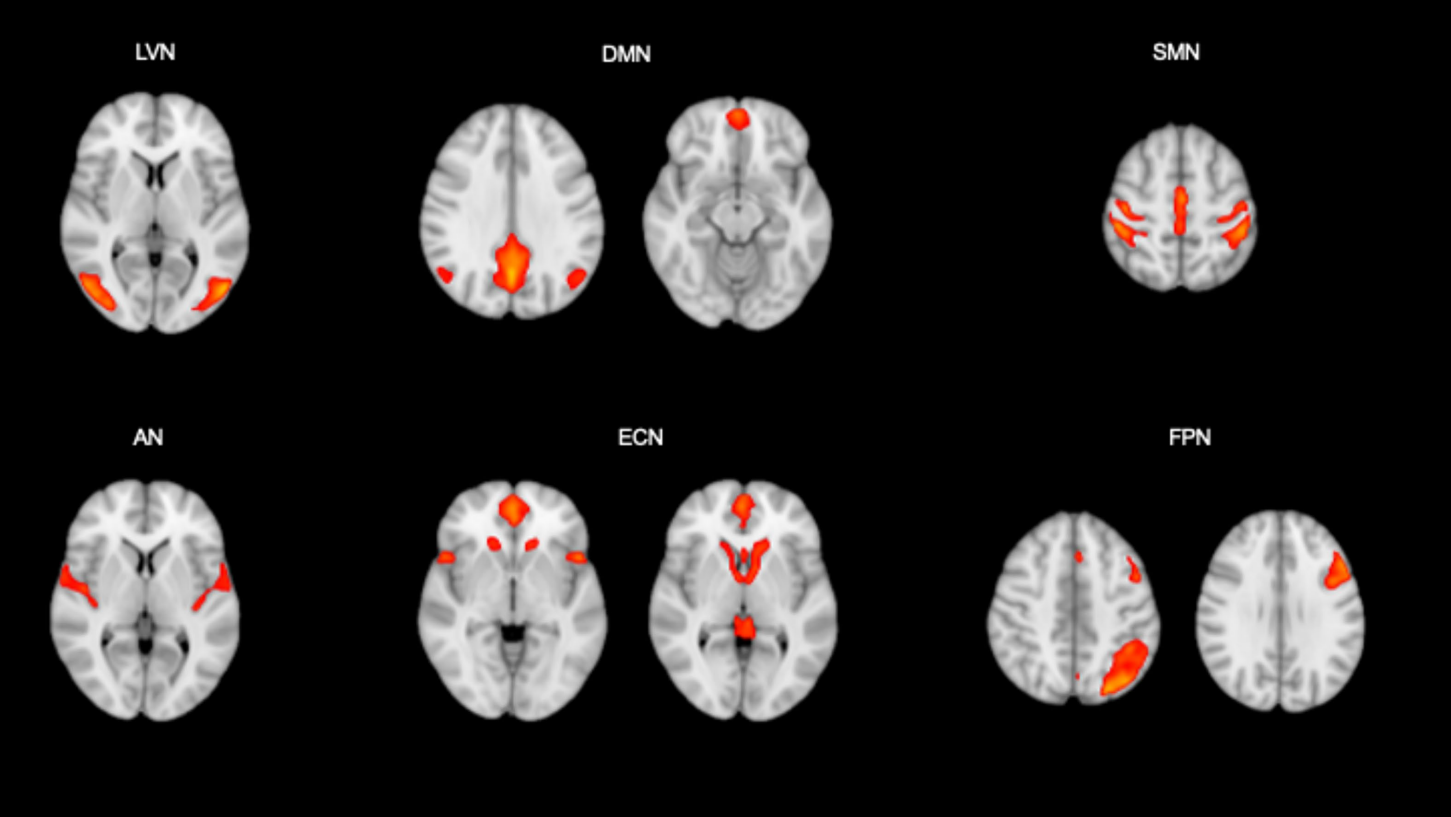
Group-averaged resting-state networks. The figure represents spatial maps of the six selected resting-state networks derived from dual regression analysis using Smith et al. (2009) network templates. Individual-level network maps (Stage 2 dual regression outputs) were first masked using binarized Smith network templates (threshold = 0.5), then averaged across patients and z-scored within each network mask. Final maps are thresholded at z > 1.96 (*p* < 0.05, two-tailed) and overlaid on a standard MNI brain template, as implemented in FSL. Networks shown include: (LVN) Lateral Visual Networks, (DMN) Default mode network, (SMN) Sensorimotor Network, (AN) Auditory Network, (ECN) Executive Control Network, (FPN) Frontoparietal Network. Colors represent z-scored signal intensity, with warmer colors indicating stronger network coherence.

Resting-state networks maps provided by Smith et al. (2009) include: three components for the visual network (medial, occipital, lateral); DMN; cerebellar network; sensorimotor network (SMN); auditory network; executive control network (ECN); and two components for the FPN (respectively corresponding to perception–somesthesis–pain, and cognition–language paradigms). The connectivity maps resulting from the dual regression procedure were normalized for residual within-subject noise prior to subsequent statistical analysis (Figure 1).

Network spatial correspondence to the template was then quantified by calculating spatial correlations between each patient’s template-derived network maps (obtained by regressing each patient’s resting-state data onto the healthy population templates via dual regression) and corresponding template networks from Smith et al. (2009), providing a measure of spatial network integrity. Correlation values range from 0 to 1. Correlations coefficients of *r* ≥ 0.5 are expected in healthy subjects (Amemiya et al., 2023), although with different inter-individual variability for specific network (Amemiya et al., 2023); correlation coefficients of *r* ≥ 0.25 are generally considered as representing neurobiologically meaningful network correspondence (Smith et al., 2009). Higher correlation values indicate better preserved network spatial organization, while lower values suggest network disruption due to lesion effects. Based on previous literature, we focused our analyses on the following networks: lateral visual network (LVN), involved in cognition and visuospatial abilities; DMN; SMN; auditory network; ECN; and the cognition–language component of the FPN (Smith et al., 2009). The occipital visual network was used as a quality control measure, as no patients had lesions located in the occipital regions, and template similarity of visual occipital network was not expected to show correlations with neuropsychological variables. Correlation coefficients for our networks are reported in Table 1. Given the low coefficient of the cerebellar network, despite no patients having resection cavities or residual lesions located in the cerebellum, this network was excluded from further analysis due to concerns in terms of signal loss and susceptibility artifacts. Indeed, respiratory-induced signal fluctuations tend to increase in the inferior direction and are particularly higher in the cerebellum (van der Zwaag et al., 2015).

**Table 1:** Network-to-template correspondence scores.

Network	Mean (SD)	Min; Max
Lateral visual network	0.25 (0.07)	0.12; 0.44
Default-mode network	0.38 (0.08)	0.22; 0.54
Sensorimotor network	0.22 (0.05)	0.12; 0.36
Auditory network	0.26 (0.07)	0.12; 0.43
Executive control network	0.24 (0.05)	0.14; 0.35
Fronto-parietal network	0.25 (0.07)	0.10; 0.41

### Statistical analyses

Performance scores from individual cognitive tests were converted to T-scores (i.e., standardized scores with a mean of 50 and a SD of 10). Composite scores were then calculated for six cognitive domains by averaging the T-scores within each domain. The BVMT-R, including both learning and delayed recall trials, was used as a measure of visual learning and memory. Verbal learning and memory were evaluated with the RAVLT, incorporating learning and delayed recall components. Visuospatial and perceptual abilities were measured using TMT Part A and the RCFT, copy condition. Language functioning was assessed through the BNT, phonemic fluency tasks (phonemic fluency and semantic fluency: animals), and the CWIT, conditions 1. Processing speed and attention were evaluated using the Coding subtest and Digit Span Forward from the WAIS-IV. Executive function was measured with TMT Part B and the CWIT, conditions 3–4. Non-native Swedish speakers were excluded from language-dependent tests but retained in analyses when nonverbal data was available.

First, spatial network re-organization was tested for associations with domain-specific cognitive performance by fitting separate linear regression models for each of the selected networks: LVN, DMN, SMN, ECN, auditory network, and FPN; the medial occipital visual network was used as a control network. All analyses were corrected for age, gender, and time since surgery, to account for potential progressive compensatory effects and/or delayed treatment effects. Bonferroni correction for multiple testing was applied, with a significance threshold of *p* = 0.0014 (0.05/6 cognitive domains/6 networks). We further corrected for radiotherapy status (i.e., whether the patient had been treated with radiotherapy or not) as a sensitivity analysis.

Next, to identify which cognitive domains best predicted subjective experience of mental fatigue in our patient sample, we conducted separate linear regression analyses examining the relationship between each cognitive test and MFS scores. All models included age and sex as covariates. To control for multiple comparisons, we applied a Bonferroni correction and set the significance threshold at *p* < 0.008 (0.05/6 cognitive domains).

## Results

### Participants

All patients were classified according to WHO 2021 classification of tumors of the Central Nervous System (Board WCoTE, 2021), where all had confirmed *IDH* mutated glioma from the primary surgery. The tumors were equally distributed between hemispheres (50.8% right), and 66.7% were located in the frontal lobe. There were 58.7% oligodendrogliomas, while 26 were classified as *IDH*-mutated astrocytomas. The distribution of photon vs. proton radiotherapy was comparable in the sample; only six patients did not undergo any radiotherapy treatment. See Table 2 for a summary of demographics and clinical characteristics of the sample.

**Table 2:** Sample characteristics.

Variable	Mean (SD)
Age at follow-up (years)	48.1 (11.3)
Time since surgery (years)	8.4 (3.7)
HADS depression	3.4 (3.5)
HADS anxiety	6.7 (4.6)
MFS	13.1 (7.6)

Variable	Count (percentage)
Sex (female)	29 (46.0%)
Hemisphere (right)	32 (50.8%)
Lobe (frontal)	42 (66.7%)
Lobe (parietal)	9 (14.3%)
Lobe (temporal)	8 (12.7%)
Lobe (insular)	4 (6.3%)
Molecular diagnosis (astrocytoma)	26 (41.3%)
Molecular diagnosis (oligodendroglioma)	37 (58.7%)
WHO grade 2	37 (58.7%)
WHO grade 3 + 4	24 + 2 (41.3%)
Radiotherapy (photon)	28 (44.4%)
Radiotherapy (proton)	29 (46.0%)

### Neuropsychological assessment

Table 3 presents the results from the neuropsychological assessment. As noted, the number of participants varies slightly across tests. For patients with a non-native language background, most language-based tests could not be administered. In a few cases, some tests were unavailable, and in one instance, only a few tests could be completed due to severe mental fatigue. Overall, the range of patients with at least one score available on each domain ranged from 59 to 61 (Table 3).

**Table 3:** Summary of neuropsychological assessment.

Cognitive domain	Test	*N*	Mean *T*-values (SD)
Visual learning and memory	BVMT-R, learning	59	47.98 (13.3)
	BVMT-R, recall	59	50.92 (13.4)
Verbal learning and memory	RAVLT, learning	60	48.08 (13.3)
	RAVLT, delayed recall	61	51.62 (11.6)
Visuospatial and perception abilities	TMT, part A	60	49.47 (10.9)
	RCFT, copying	52	45.90 (8.3)
Language	BNT	56	45.39 (12.6)
	FAS	61	45.84 (15.2)
	Animal naming	52	53.90 (16.4)
	D-KEFS, CWIT, condition 1	57	42.82 (10.3)
Processing speed and attention	WAIS-IV, coding	60	45.72 (9.5)
	WAIS-IV, digit span forward	61	48.31 (9.9)
Executive function	TMT, part B	60	45.10 (12.9)
	D-KEFS, CWIT, condition 3	56	46.16 (15.9)
	D-KEFS, CWIT, condition 4	56	45.57 (13.1)

BNT, Boston Naming Test; BVMT-R, Brief Visuospatial Memory Test–Revised; CWIT, Color Word Interference Test; D-KEFS, Delis-Kaplan Executive Function System; FAS, phonemic fluency; RAVLT, Rey Auditory Verbal Learning Test; RCFT, Rey Complex Figure Test; TMT, Trail Making Test; WAIS, Wechsler Adult Intelligence Scale.

### Associations between cognitive performance and network-template similarity

Executive function showed statistically significant associations with network-template similarity of the auditory network (*p* < 0.001; B coefficient = 86.39; S.E. = 24.44; standard B = 0.429; 95% C.I. = 37.41, 135.36), ECN (*p* = 0.001; B coefficient = 103.76; S.E. = 29.86; standard B = 0.424; 95% C.I. = 43.90, 163.61) and FPN (*p* < 0.001; B coefficient = 78.48; S.E. = 19.40; standard B = 0.466; 95% C.I. = 39.61, 117.35), surviving correction for multiple testing. Processing speed/attention and language functions were significantly associated with network-template similarity of the ECN (*p* = 0.001; B coefficient = 68.64; S.E. = 18.93; standard B = 0.454; 95% C.I. = 30.71, 106.57). Finally, visual learning and memory were associated with network-template similarity of the DMN (*p* < 0.001; B coefficient = 70.95; S.E. = 19.52; standard B = 0.418; 95% C.I. = 31.81, 110.10) (Table 4). All results were unchanged when further correcting for radiotherapy status.

**Table 4:** Network-template similarity and cognitive performance (*R*^2^).

Cognitive domain	AUD	DMN	ECN	FPN	LVN	SMN
Executive function	0.185**	0.072*	0.183**	0.229**	0.084*	0.114*
Processing speed and attention	0.148*	0.071*	0.193**	0.141*	0.055	0.010*
Language	0.085*	0.009	0.174**	0.118*	0.036	0.011
Visual learning and memory	0.059	0.196*	0.064	0.127*	0.013	0.012
Verbal learning and memory	0.144*	0.212	0.056	0.068*	0.042	0.036
Visuospatial and perceptual abilities	0.147*	0.045*	0.154*	0.084*	0.122*	0.067*

AUD, auditory network; DMN, default-mode network; ECN, executive control network; FPN, fronto-parietal network; LVN, lateral visual network; SMN, sensorimotor network. **Significant at the corrected threshold of *p* < 0.0014. *Significant at the uncorrected threshold of *p* < 0.05.

**Figure 2:**
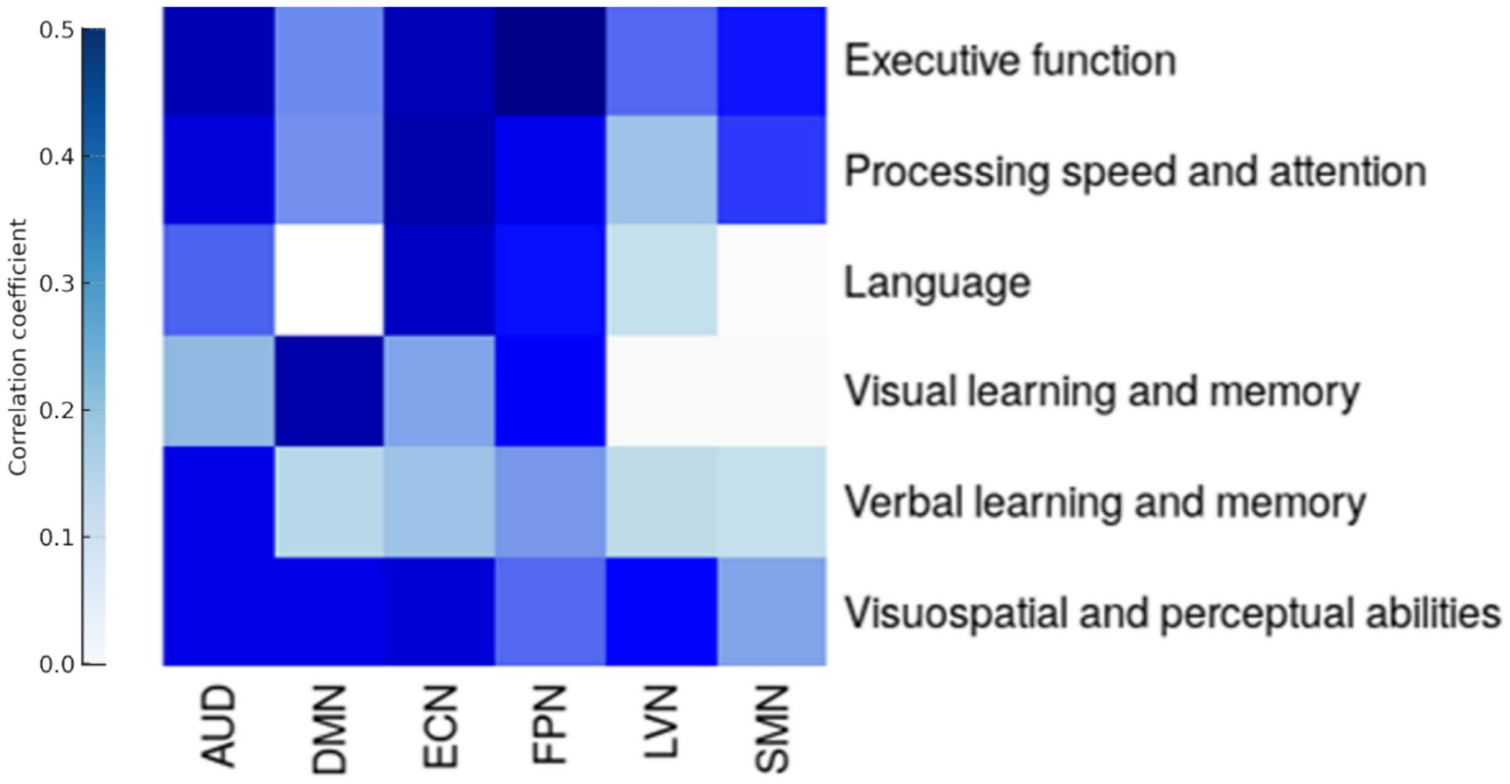
Association between network-template similarity and cognitive performance. The heatmap summarizes the correlations between cognitive performance and network-template similarity of tested networks, with darker blue corresponding to larger correlation coefficients. AUD, auditory network; DMN, default-mode network; ECN, executive control network; FPN, fronto-parietal network; LVN, lateral visual network; SMN, sensorimotor network.

As expected, network-template similarity of the medial visual network (control network) was not significantly associated with either executive functions (*p* = 0.507; *r* = 0.008), processing speed and attention (*p* = 0.132; *r*^2^ = 0.040), language functions (*p* = 0.505; *r*^2^ = 0.008), visual learning and memory (*p* = 0.933; *r*^2^ = 0.001), verbal learning and memory (*p* = 0.057; *r*^2^ = 0.063), nor visuospatial and perceptual abilities (*p* = 0.184; *r*^2^ = 0.032). A summary of all correlations can be found in Table 4 and Figure 2. Individual scatterplots are reported in Supplementary Figure S1.

### Associations between cognitive performance and mental fatigue

**Figure 3:**
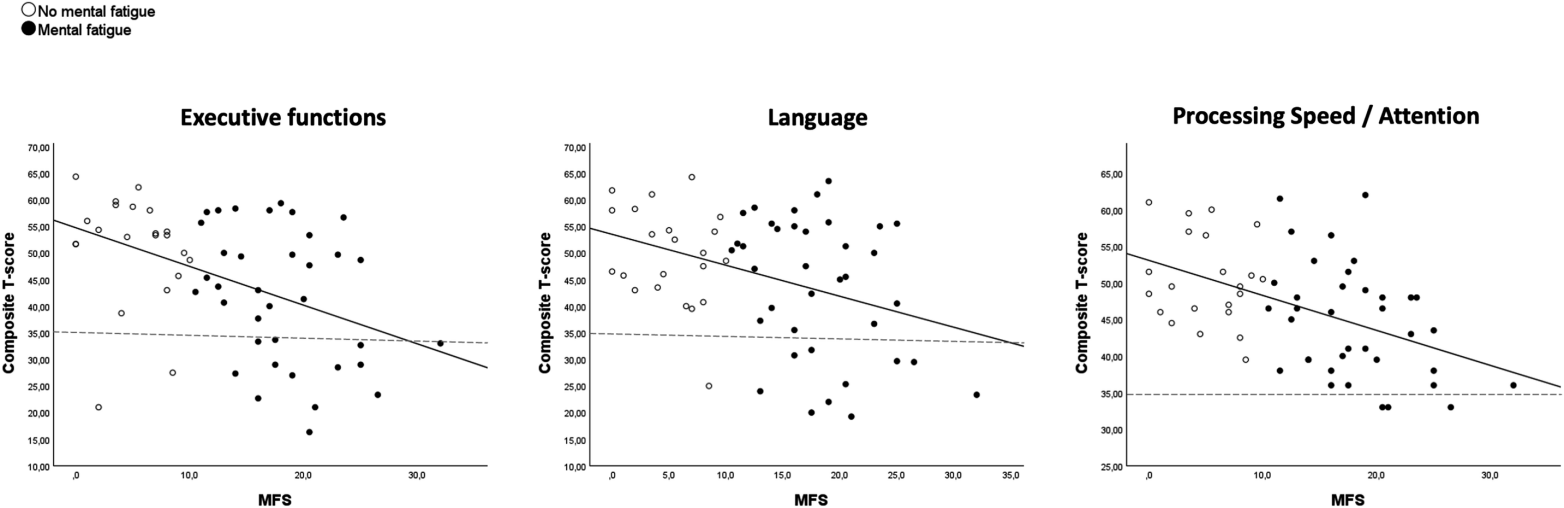
Correlations between executive function performance and mental fatigue. The scatter plots represent associations between MFS (X-axis) and cognitive performance expressed as composite *T*-values (Y-axis). Black circles represent patients with MFS scores equal to or above 10.5, indicative of mental fatigue. The dotted line represents *T*-values of 1.5 standard deviations below the mean. Executive function was measured with Trail Making Test Part B and D-KEFS CWIT tasks 3 and 4. Language functioning was assessed through the Boston Naming Test, phonemic fluency tasks (FAS test and animal naming), and the D-KEFS CWIT tasks 1. Processing speed and attention were evaluated using the Coding subtest from the Wechsler Adult Intelligence Scale and Digit Span Forward.

Statistically significant associations were found between MFS and performance on speed/attention (*p* < 0.001; *r*^2^ = −0.201; B coefficient = −0.512; S.E. = 0.136; standard B = −0.512; 95% C.I. = − 0.785, −0.239), executive functions (*p* < 0.001; *r*^2^ = −0.191; B coefficient = −0.754; S.E. = 0.220; standard B = −0.465; 95% C.I. = − 1.196, −0.313), and language (*p* = 0.003; *r*^2^ = −0.145; B coefficient = −0.646; S.E. = 0.219; standard B = −0.413; 95% C.I. = − 1.086, −0.206) (Figure 3). No associations between the MFS score and visual learning and memory (*p* = 0.054; *r*^2^ = −0.068), verbal learning and memory (*p* = 0.025; *r*^2^ = −0.088), and visuospatial and perceptual abilities (*p* = 0.102; *r*^2^ = −0.049) were found.

A notable pattern emerged when examining the relationship between cognitive performance on the executive and language domains, and mental fatigue. Nearly all patients without mental fatigue (MFS scores below 10.5) demonstrated average composite T-scores of 35 or higher on cognitive performance, corresponding to performance no lower than 1.5 standard deviations (SD) below the normative mean. In contrast, patients experiencing mental fatigue displayed a much broader range of cognitive performance, with T-scores spanning from as low as 3.4 SD below the mean, up to the same high levels observed in patients without mental fatigue. This pattern suggests that while impaired cognitive function appears to be reliably related to mental fatigue, a substantial subset of patients reported mental fatigue despite maintaining normal cognitive performance levels. This dissociation between objective cognitive abilities and subjective fatigue experience indicates that additional factors beyond cognitive impairment contribute to the development of mental fatigue symptoms.

Based on this pattern, we categorized patients into three groups for executive function and language performance (Table 5): those with preserved executive/language performance (T > 35) and no mental fatigue; patients with preserved executive/language performance (T > 35) but with mental fatigue (MFS ≥ 10.5); patients with impaired executive/language performance (T ≤ 35) and mental fatigue. For the attention and processing speed domain, only a small subset of patients (*n* = 4) who reported mental fatigue (MFS scores ≥10.5) also demonstrated impaired cognitive performance (>1.5 SD below the mean); therefore, this analysis was not performed.

**Table 5:** Subgroups based on cognitive performance and mental fatigue.

Performance	Mental fatigue	No mental fatigue
Executive function		
Preserved (T > 35)	23	20
Impaired (T < 35)	13	–
Language		
Preserved (T > 35)	27	21
Impaired (T < 35)	10	–

Discriminant analyses were carried out to explore which variables could better predict assignment to the fatigue-cognition profiles previously defined. Two discriminant analyses were carried out: one included age, sex, psychological variables (depression and anxiety) and network-template similarity scores; the other included sex, age, and tumor-related characteristics. A stepwise method using Wilks’ lambda was applied with entry criterion of *p* < 0.05 and removal criterion of *p* < 0.10.

### Discriminant analysis

**Figure 4:**
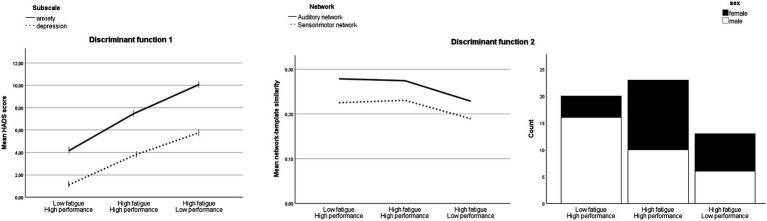
Discriminant analysis of cognitive-fatigue profiles. The figure illustrates the individual components of the two discriminant functions that emerged from the analysis of executive function and fatigue profiles. The first discriminant function was primarily driven by HADS (Hospital Anxiety and Depression Scale) depression and anxiety subscale scores (left panel; line plot), while the second function was determined by network-template similarity of the sensorimotor and auditory networks (middle panel; line plot) and sex distribution (right panel; histogram).

Two discriminatory functions were identified, predicting group assignment to the fatigue-executive function profiles. The first function, explaining 81.4% of the variance with a Wilk’s lambda of *p* < 0.001, primarily distinguished groups based on anxiety and depression scores (positive loadings of 0.752 and 0.609, respectively) (Figure 4). The second function, explaining an additional 18.6% of the variance with a Wilk’s lambda of *p* = 0.035, primarily distinguished groups based on network-template similarity of the sensorimotor and auditory networks, and sex (positive loadings of 0.759, 0.650, and 0.615, respectively) (Figure 4). No other loading above 0.50 were identified.

Function 1 (including depression and anxiety measures) could discriminate between patients with and without mental fatigue (discriminant scores significantly different between groups with *p* < 0.004); however, it could not distinguish patients with and without impairments on executive function performance within the mental fatigue group (*p* = 0.077). While patients who reported experiencing mental fatigue had higher depression scores compared with patients without mental fatigue (*p* < 0.001), no significant differences were observed within patients with mental fatigue, with or without difficulties in the executive function domain (*p* = 0.423). Similarly, while differences in anxiety could be observed between patients with and without mental fatigue (*p* < 0.009), no significant differences in anxiety scores were detected between patients with and without impairments on executive function performance within the mental fatigue group (*p* = 0.207).

Function 2 could discriminate between patients with and without executive function impairments within the mental fatigue group (*p* = 0.022), and could also discriminate between patients with and without mental fatigue in the high executive performance group (*p* = 0.029). When looking at the specific score components, patients with high executive functions did not differ significantly on network-template similarity of the auditory (*p* = 0.345) or sensorimotor network (*p* = 0.411). However, within the group experiencing mental fatigue, patients with low executive function had significantly lower network-template similarity on the sensorimotor (*p* = 0.007) and auditory (*p* = 0.018) networks compared with patients with high executive function and experiencing mental fatigue. Sex distribution was different between patients with and without mental fatigue but not between different levels of executive functions, with most of the patients without mental fatigue being male (16 out of 20), compared to a roughly equal distribution in the mental fatigue group.

Two discriminatory functions were also identified when predicting group assignment to the fatigue-language profiles. However, while the first function explained 90.1% of the variance, the second function only explained an additional 4.0% of the variance, with a non-significant Wilk’s lambda of 0.136, and was thus not considered. The first, significant function primarily distinguished groups based on depression scores (positive loading of 0.696). Similar to fatigue-executive function profiling, however, while the analysis could discriminate between patients with and without mental fatigue (discriminant scores significantly different between groups with *p* < 0.001), it could not distinguish patients with and without impairments on language tests performance within the mental fatigue group (*p* = 0.121). Accordingly, while patients who experienced mental fatigue had higher depression scores compared with patients without mental fatigue (*p* < 0.001), no significant differences were observed within patients with mental fatigue, with or without difficulties in the language domain.

The discriminant analysis examining tumor-related characteristics failed to identify meaningful discriminatory patterns between cognitive-fatigue groups. Only sex emerged as a significant predictor for distinguishing between the fatigue-executive and fatigue-language groups, indicating that tumor-related variables do not substantially contribute to cognitive-fatigue profile determination.

## Discussion

To our knowledge, this is the largest rs-fMRI study of IDH-mutated glioma survivors with a follow up of more than 5 years, revealing that preserved large-scale network integrity is important for maintaining long-term cognitive functions. Network-template similarity across multiple brain networks correlated with domain-specific cognitive performance, with auditory network, ECN, and FPN being the most relevant across cognitive domains. Executive function, language, and processing speed and attention emerged as the most relevant cognitive domains associated with patient-reported experience of mental fatigue. Executive function-fatigue profiles could be characterized into three groups: (1) patients with preserved executive function and no mental fatigue were predominantly male, exhibited low anxiety and depression scores, and maintained higher network-template similarity in auditory and sensorimotor networks; (2) patients with preserved executive function but experiencing mental fatigue showed equal gender distribution, higher depressive scores, and were more likely to have mild to moderate anxiety symptoms, but retained comparable auditory and sensorimotor network-template similarity to the non-fatigued group; (3) patients with impaired executive function and mental fatigue demonstrated equal gender distribution, the highest depression scores, more likely to have clinical anxiety, and the most compromised network-template similarity in auditory and sensorimotor networks.

### Network-cognition associations

Higher network-template similarity was associated with better cognitive performance, indicating that network organization patterns similar to those in healthy populations may relate to better cognitive outcomes. In particular, template similarity of FPN, ECN, and auditory networks emerged as associated with the majority (4 out of 6) of the cognitive domains tested in our sample of long-term *IDH*-mutated glioma patients, consistent with previous literature demonstrating that cognitive function depends on the integrity of multiple interconnected networks rather than isolated systems (van Lingen et al., 2023; Liu et al., 2019; Yang et al., 2021; Ng et al., 2023). Alterations in the FPN and ECN have also previously been reported in glioma patients, partly relating to cognitive alterations (van Lingen et al., 2023; Liu et al., 2019; Yang et al., 2021). These findings suggest that cognitive rehabilitation interventions should target shared higher-order control processes like working memory, cognitive control, and attention regulation, potentially leveraging compensatory neural plasticity and behavioral adaptation.

### Cognitive and neural profiles

We investigated whether cognitive performance was linked to subjective mental fatigue. Executive function, processing speed and attention, and language performance correlated significantly with mental fatigue scores, while visual and verbal memory, and visuospatial abilities did not. Additionally, we identified distinct patient subgroups based on fatigue-cognition profiles. Patients without mental fatigue performed in the normal range on executive and language tests, while patients with mental fatigue showed broad performance ranging from severely impaired (>3 SD below mean performance) to normal levels. This dissociation between objective cognitive abilities and subjective mental fatigue indicates that factors beyond cognitive impairment may contribute to mental fatigue symptoms.

Anxiety, depression, and sex emerged as primary predictors of patient-reported mental fatigue, while auditory network and SMN network-template similarity predicted executive function performance. Patients with both executive function impairment and mental fatigue exhibited high depression and anxiety scores approaching clinical thresholds, and reduced network-template similarity. In contrast, patients without mental fatigue and with preserved executive function showed low depression and anxiety scores, were predominantly male, and had higher network-template similarity. Patients with preserved executive function but persistent mental fatigue showed a mixed profile, with network integrity comparable to unimpaired patients but elevated anxiety and depression. This pattern reveals that psychological symptoms (depression/anxiety) primarily drive mental fatigue regardless of executive performance, while brain network-template similarity is specifically associated with executive function capacity within patients experiencing fatigue.

The relationship between depression, anxiety and fatigue is well-established (Mozuraityte et al., 2023), with fatigue being a known component of major depressive disorder (Eggart et al., 2023). Several brain regions and networks have been associated with mental fatigue, including the DMN, the ECN, salience network, and insula (Pessiglione et al., 2025; Matuz et al., 2024; Darnai et al., 2023; Anderson et al., 2019; Trojsi et al., 2023; Wylie et al., 2020). While not typically part of the “fatigue network” (Wylie et al., 2020), auditory processing has also been linked to mental fatigue (Moore et al., 2017). However, most of the research on mental fatigue has been conducted in different clinical populations [e.g., traumatic brain injury (Bruijel et al., 2022; Ramage et al., 2018), multiple sclerosis (Chen et al., 2020; Langley et al., 2023)], complicating the identification of fatigue-specific versus condition-specific brain patterns. Importantly, SMN connectivity has been associated with both anxiety (Bouziane et al., 2022) and depression (Ray et al., 2021), and auditory and multisensory stimulations have been proposed as treatments to modulate mood and alleviate depression and anxiety (Canbeyli, 2013), possibly through sensorimotor gating modulation (Storozheva et al., 2021).

Our cross-sectional design precludes determining whether heightened anxiety and depression precede or result from cognitive dysfunction. Similarly, the role of preserved auditory and sensorimotor network integrity in maintaining executive function among fatigued patients remains unclear and cannot establish causality. Future longitudinal studies examining mood, cognitive function, and neural networks over time are essential to explore causal relationships and understand how these factors influence each other throughout the disease trajectory.

### Clinical implications

These findings suggest that cognitive rehabilitation in glioma survivors should prioritize higher-order functions such as attention, working memory, and executive control, supported by targeted cognitive-behavioral therapies and metacognitive strategies. Because mental fatigue was closely linked to depression and anxiety, routine screening and treatment of mood symptoms should be considered an integral part of fatigue management.

Although rs-fMRI is not yet a clinical stratification tool, network-level measures may in the future complement neuropsychological testing by providing objective markers of recovery and guiding personalized rehabilitation. Moreover, the involvement of auditory and sensorimotor networks highlights opportunities to explore non-invasive approaches such as music therapy, rhythmic auditory stimulation, or exercise-based interventions as adjunctive strategies to improve resilience and quality of life.

### Limitations

The cross-sectional design with variable follow-up times (5–21 years post-surgery) limits our ability to draw causal inferences about the relationship between preservation of network spatial organization and cognitive outcomes. The lack of longitudinal data prevents assessment of how these relationships evolve over time. While the absence of healthy control data restricts our ability to establish normative baselines for network-template similarity measures in this population, the use of a well-established atlas mitigated this limitation. Our sample, while larger than most previous studies in this field, consisted exclusively of *IDH*-mutated glioma patients, and is therefore suited to answer research questions in the presence of a slow-growing malignant brain tumors with the preferential location being in the frontal, temporal and insular regions. As part of clinical routine, the treatment provided in these cases was not uniform and adds heterogeneity, although most of the patients received resective surgery and oncological treatment.

Methodological considerations include the use of templates derived from healthy populations (Smith et al., 2009) for assessing network-template similarity in a clinical sample, which may not fully capture the altered network topography present in brain tumor patients. Indeed, the average network correspondence to the template was on the low end of the range, spanning from 0.24 to 0.38, where 0.25 is considered meaningful for neurobiological effects (Smith et al., 2009). However, deviations from healthy templates are expected in a clinical population of patients who underwent brain surgery for tumor resection. Moreover, the specificity of our findings is supported by the use of the medial visual network as a negative control. As predicted, this network showed no significant association with executive function (*p* = 0.169), indicating that our approach can distinguish between networks that are and are not expected to relate to cognitive performance.

Finally, we cannot rule out the influence of unmeasured factors such as medication effects, comorbidities, or individual differences in cognitive reserve that may modulate the observed relationships between brain networks and cognitive performance. Indeed, while our sample reflects the natural distribution of a rare, histologically homogeneous tumor type observed in real-world clinical settings, it also introduces heterogeneity in variables that cannot be fully controlled. A substantial proportion of patients (64%) were using antiepileptic drugs (AEDs) at the time of assessment, which may influence both cognitive performance and BOLD signal dynamics; the specific agents and doses were not systematically controlled for, and future studies should examine AED type as a potential moderating variable. Similarly, corticosteroid use, if present at time of scanning, may affect network integrity metrics and should be recorded and reported in future work. The mixed treatment history of the cohort, with some patients having received radiotherapy and others not, represents a further source of variance; while radiotherapy status was included as a covariate and did not substantively alter the results, the sample size precluded a fully powered subgroup analysis. Finally, the wide age range (25–80 years), though reflective of the epidemiology of IDH-mutated gliomas and addressed through covariate correction, may introduce residual variance in baseline neuroplasticity and age-related cognitive decline that cannot be entirely eliminated in a sample of this size.

### Future directions

Future research should address several methodological limitations identified in this study. Longitudinal designs beginning pre-operatively are needed to establish causal relationships between network changes and cognitive outcomes. The inclusion of healthy control groups would also be necessary to establish normative baselines for network measures in this population.

## Conclusion

This study provides novel insights into the complex relationships between functional network to template similarity, mental fatigue, and cognitive function in patients with IDH-mutated glioma living with the disease long term. In our sample, different networks mediated the relationship between fatigue and cognition, suggesting that complex, multiple mechanisms are underlying cognitive dysfunction in these patients. In particular, our findings demonstrate that preserved spatial organization of multiple brain networks—particularly the auditory, executive control, and frontoparietal networks—is associated with better cognitive function performance in long-term glioma survivors, while networks such as the sensorimotor and auditory networks appear to play protective roles in patients experiencing fatigue symptoms, potentially serving compensatory functions that help maintain cognitive performance despite symptom burden. Customized interventions targeting depression and anxiety may therefore ameliorate fatigue symptoms in specific subsets of patients.

## Data Availability

The data analyzed in this study is subject to the following licenses/restrictions: the data that support the findings of this study are available on request. The data are not publicly available due to GDPR restrictions. Data can be requested to ASJ. A data use agreement will be requested. Requests to access these datasets should be directed to Asgeir Store Jakola, jakola.asgeir@gu.se.

## Footnotes

1
https://www.applied-statistics.de/lst.html

2
https://www.fil.ion.ucl.ac.uk/spm/software/spm12/

3
http://www.fmrib.ox.ac.uk/analysis/brainmap+rsns/
