# Supplementary material for: Relationship between functional network integrity, cognition and fatigue at long-term follow-up in patients with IDH-mutated gliomas
Source: Front Hum Neurosci. 2026 Jul 16;20:1860966. doi: 10.3389/fnhum.2026.1860966 (PMC13422171; doi:10.3389/fnhum.2026.1860966)
Supplement: Supplementary file 1 [file Data_Sheet_1.docx]

**Supplementary Material**

**Table S1. Description of the neurocognitive tests.**

| **Test** | **Description** |
| --- | --- |
| Boston Naming Test (BNT) | A confrontation naming task comprising 60 drawings of objects. The participant names each picture aloud, assessing lexical retrieval and language abilities. |
| D-KEFS Verbal Fluency; semantic fluency, and phonemic fluency | In the semantic condition, the participant generates as many words as possible within one minute from a semantic category (e.g., animals). In the **phonemic** condition, words beginning with specific letters (F, A, S) are produced within one minute each. Both tasks assess verbal fluency and processing speed; the phonemic condition also involves executive control. |
| Rey Auditory Verbal  Learning Test (RAVLT) | A list of 15 words is read aloud five times, followed by immediate recall after each trial. After a 30-minute delay, a delayed recall trial is conducted. The test measures verbal learning, recall, and retention. |
| Rey Complex figure,  (RCFT) copying | Participants reproduce a complex geometric figure while viewing the original. The task assesses visuospatial constructional skills and planning aspects of executive functioning. |
| Brief Visuospatial Memory Test-Revised (BVMT-R) | Six geometric designs are displayed for 10 seconds on three consecutive trials. After each exposure, participants reproduce the figures from memory, followed by a delayed recall trial after 25 minutes. The task measures visuospatial learning and memory. |
| Trail Making Test, (TMT)  A and B | In part A, the participant connects numbered circles in ascending order, reflecting visual scanning, processing speed, and attention.  In part B, the participant alternates between numbers and letters (1–A–2–B…), assessing cognitive flexibility/executive functioning. |
| D-KEFS Color-Word Interference Test (CWIT) | All subtests should be performed as rapidly as possible.  CWIT 1. Naming colors, measures naming speed.  CWIT 3. Naming the color of incongruently colored words, measures verbal speed and response inhibition CWIT 4. Same as 3 but alternating between color naming and reading measuring aspects of cognitive flexibility. |
| WAIS-IV, Digit Span | A series of digits is read aloud with increasing length. In the forward condition, participants repeat the digits in the same order (short-term memory and attention). In the backward condition, digits are recalled in reverse order (working memory/executive functioning). |
| WAIS IV, Coding | Participants transcribe symbols paired with numbers using a provided key within a two-minute time limit. The task measures processing speed, sustained attention, and visuomotor coordination. |

**Figure S1.**

*
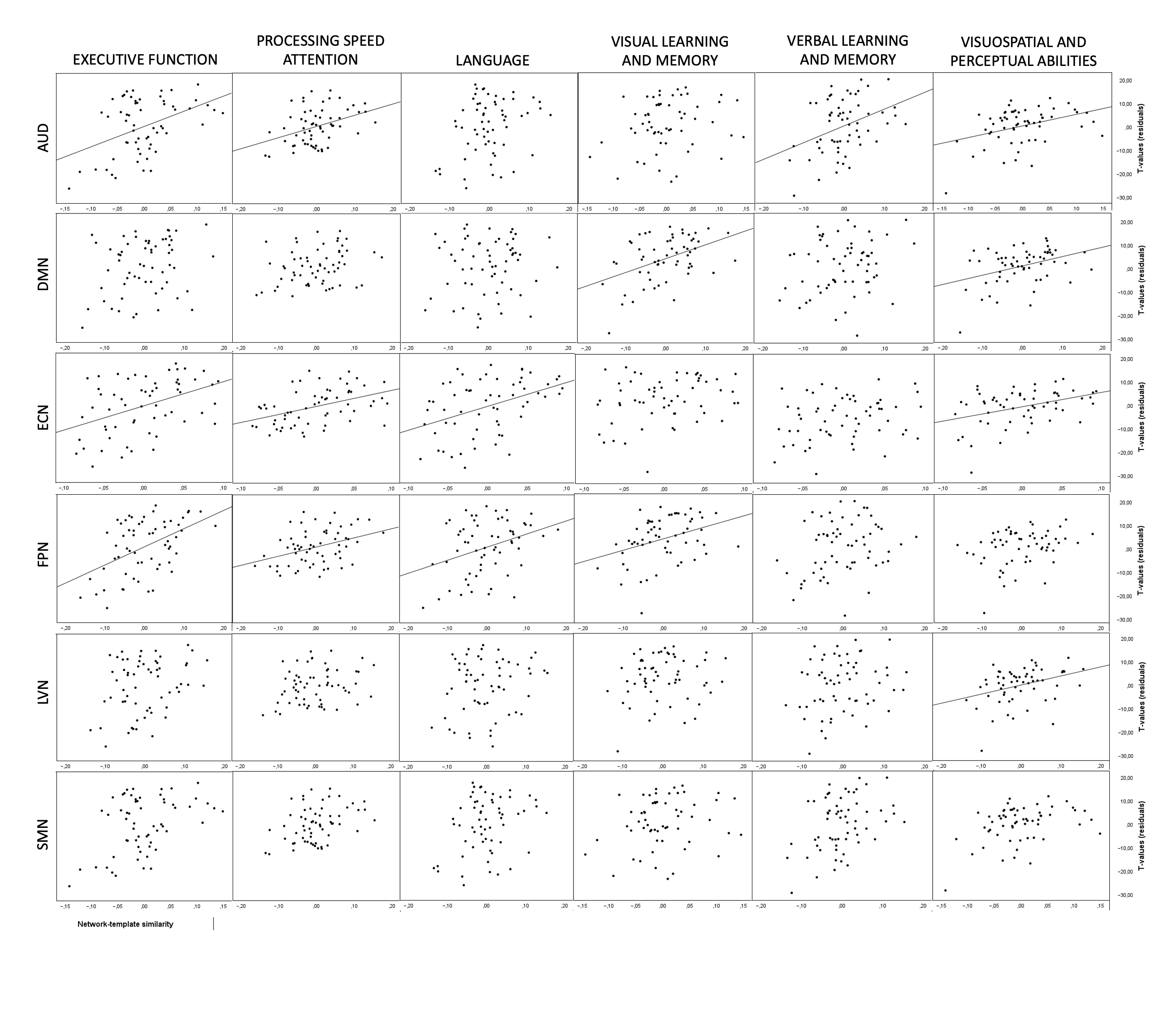
*

**Figure S1. Association between network-template similarity and cognitive performance.** The figure shows partial regression plots between cognitive performance and network-template similarity of tested networks. The y-axes represent the residuals of cognitive performance on different domains (from left to right column: executive function, processing speed/attention, language, visual learning and memory, verbal learning and memory, visuospatial and perception abilities) after removing the linear effects of the other predictor variables in the model (sex, age, time since surgery). In brief, the residuals represent the part of the dependent variable that cannot be explained by the other predictors in the regression model. The x-axes report the network-template similarity of the tested network (from top to bottom: auditory network (AUD), default-mode network (DMN), executive control network (ECN), fronto-parietal network (FPN), lateral visual network (LVN) and sensorimotor network (SMN).
